# Supplementary material for: Ultrasonic-Assisted Synthesis of Benzofuran Appended Oxadiazole Molecules as Tyrosinase Inhibitors: Mechanistic Approach through Enzyme Inhibition, Molecular Docking, Chemoinformatics, ADMET and Drug-Likeness Studies
Source: Int J Mol Sci. 2022 Sep 19;23(18):10979. doi: 10.3390/ijms231810979 (PMC9500974; doi:10.3390/ijms231810979)
Supplement: Supplementary file 1 [file ijms-23-10979-s001.zip › ijms-1880450-supplementary.pdf]

# Ultrasonic-Assisted Synthesis of Benzofuran Appended Oxadiazole Molecules as Tyrosinase Inhibitors: Mechanistic Approach through Enzyme Inhibition, Molecular Docking, Chemoinformatics, ADMET and Drug-Likeness Studies

**Ali Irfan [1], Ameer Fawad Zahoor [1,*], Shagufta Kamal [2], Mubashir Hassan [3] and Andrzej Kloczkowski[3,4,*]**

## SUPPLEMENTARY INFORMATION

### Spectral analysis of compounds 5a-5j

**2-((5-(5-Bromobenzofuran-2-yl)-1,3,4-oxadiazol-2-yl)thio)-N-(2-fluorophenyl)acetamide (5a)**

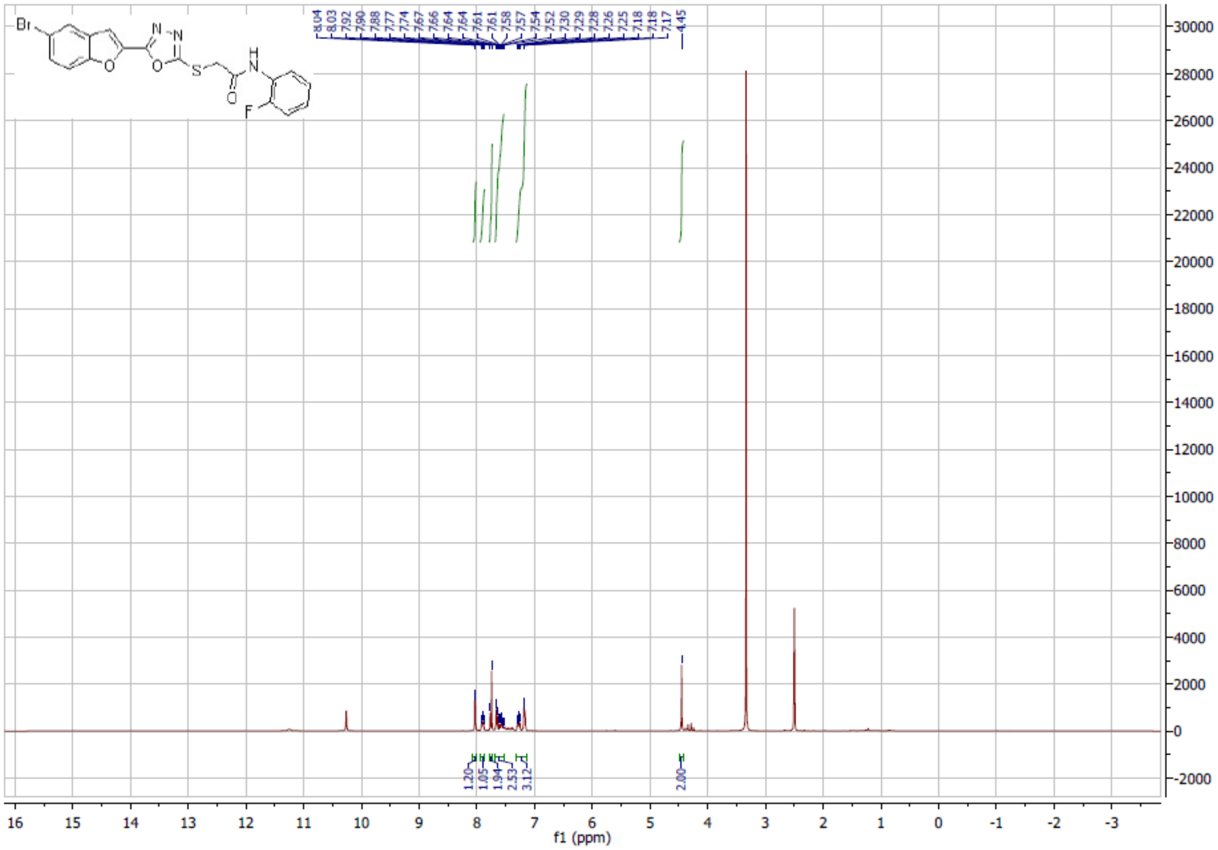

**Figure S1.** $^1$H NMR spectrum of compound **5a**

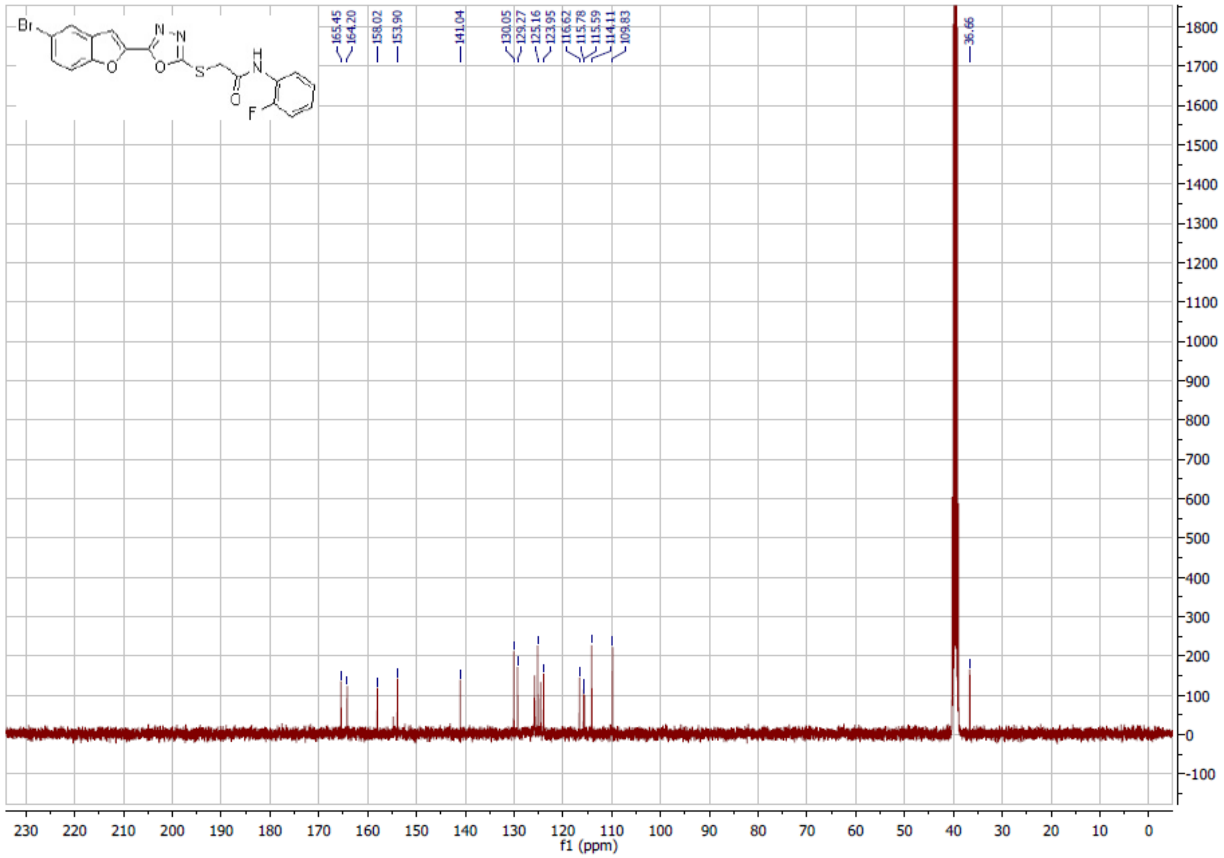


**Figure S2.**$^{13}$C NMR spectrum of compound **5a**

**2-((5-(5-Bromobenzofuran-2-yl)-1,3,4-oxadiazol-2-yl)thio)-N-(2-chlorophenyl)acetamide (5b)**

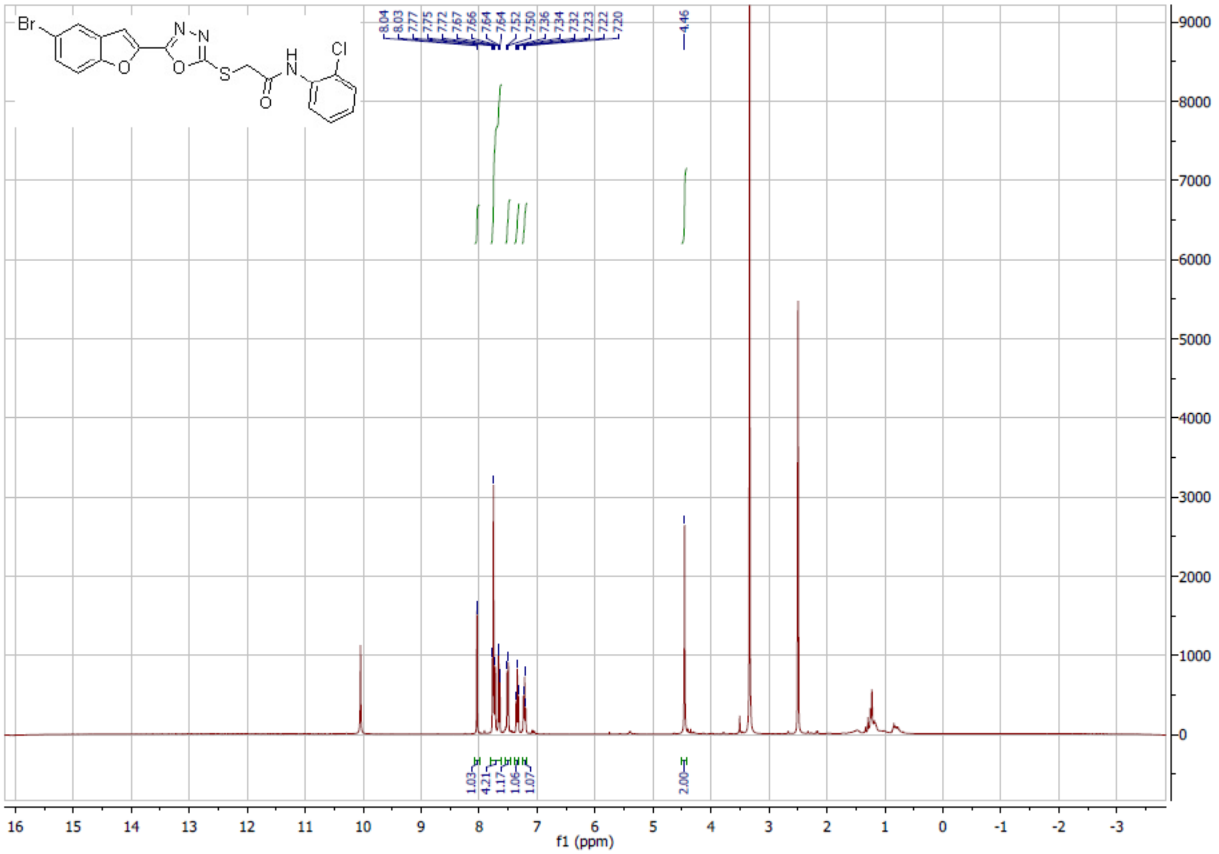


**Figure S3.**$^{1}$H NMR spectrum of compound **5b**

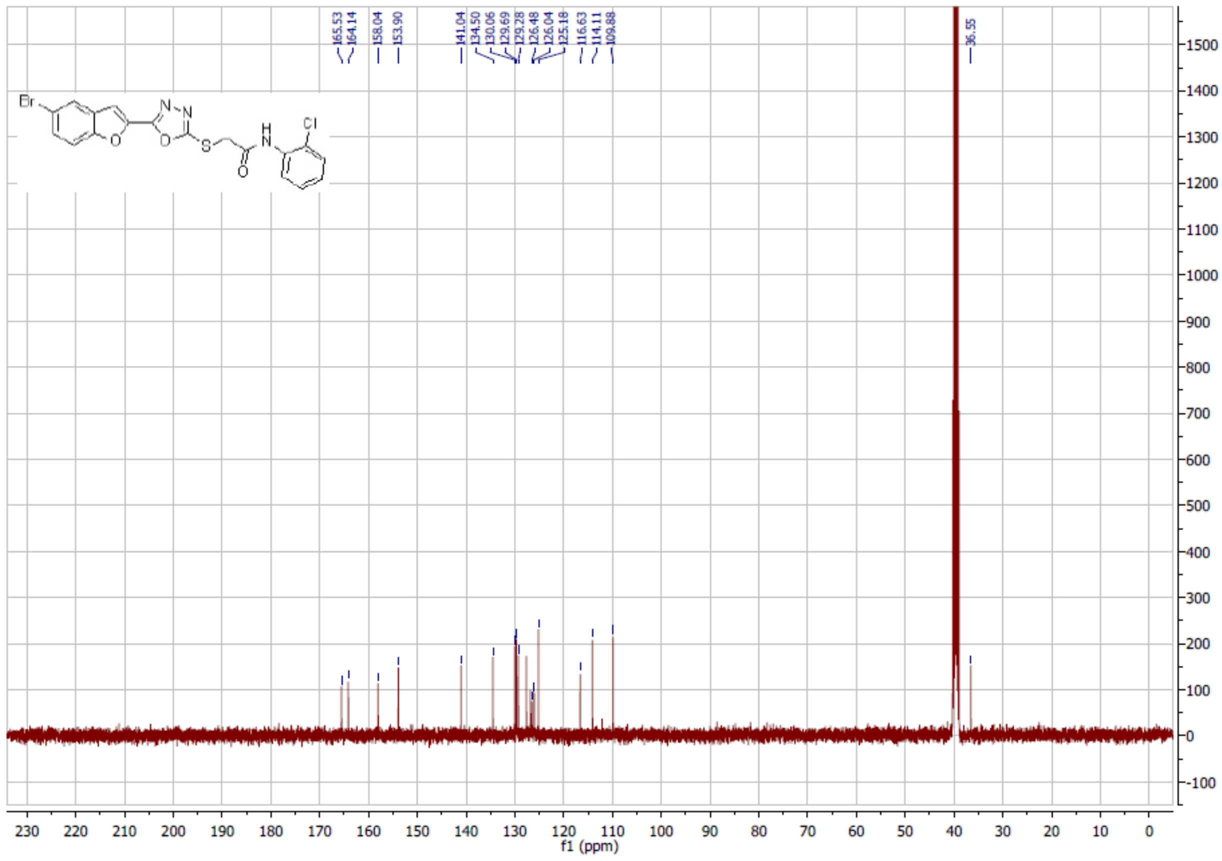


**Figure S4.**$^{13}$C NMR spectrum of compound **5b**

**2-((5-(5-Bromobenzofuran-2-yl)-1,3,4-oxadiazol-2-yl)thio)-*N*-(3,4-dichlorophenyl)acetamide (5c)**

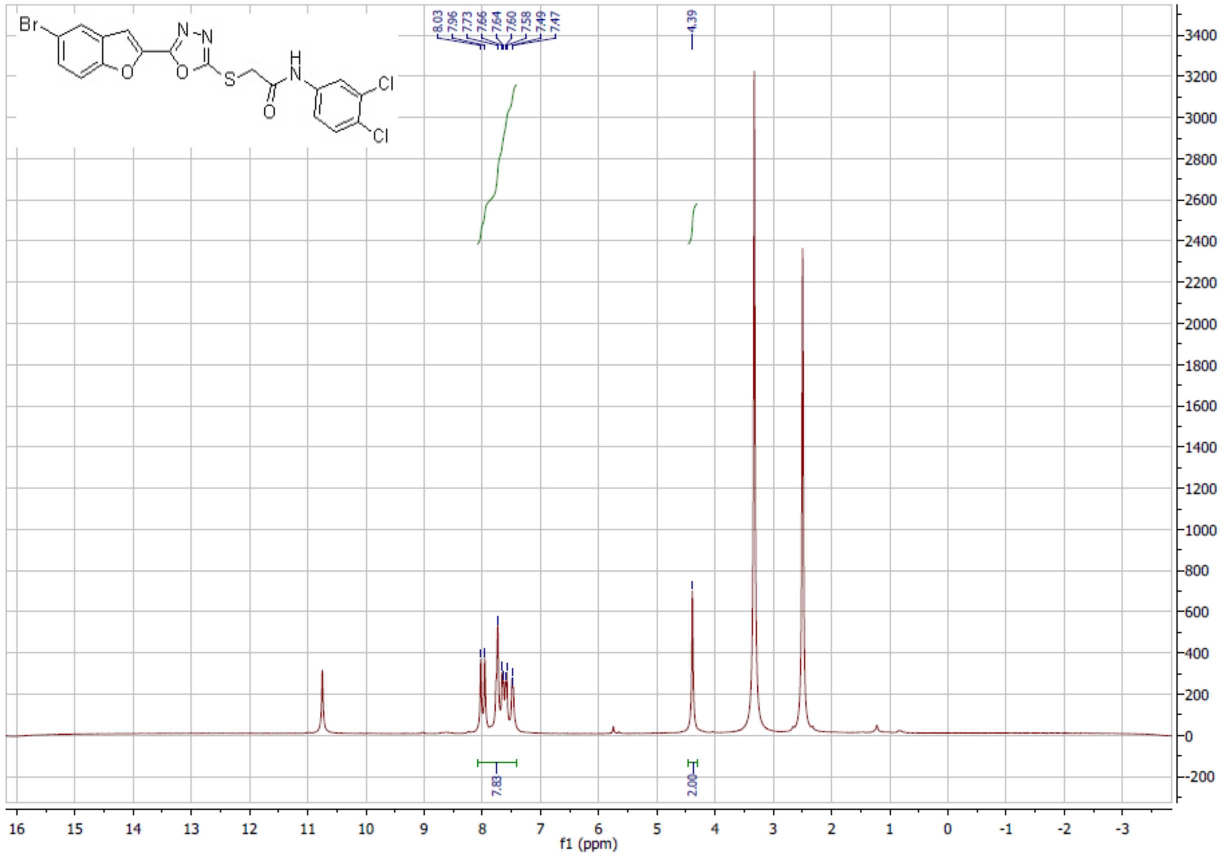

**Figure S5.** $^{1}$H NMR spectrum of compound **5c**

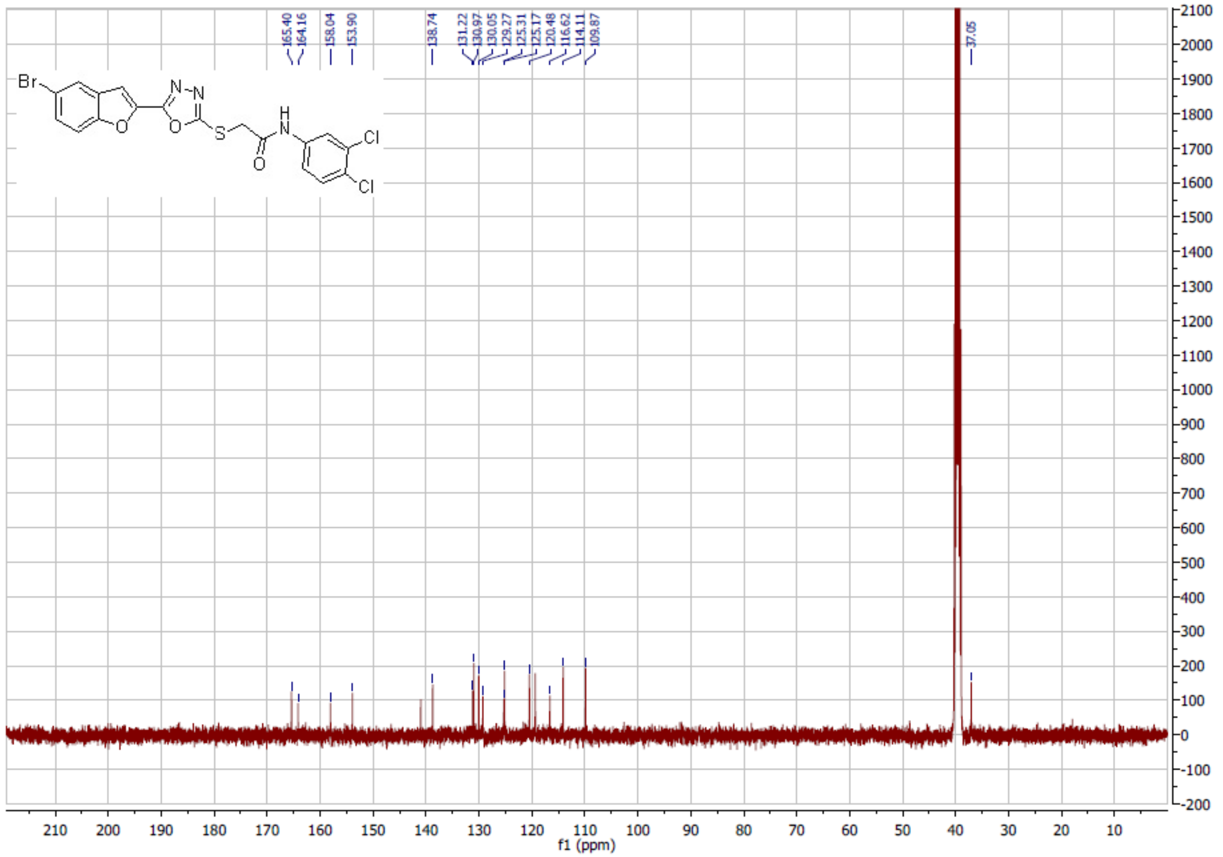

**Figure S6.**$^{13}$C NMR spectrum of compound **5c**

**2-((5-(5-bromobenzofuran-2-yl)-1,3,4-oxadiazol-2-yl)thio)-N-(4-fluorophenyl)acetamide (5d)**

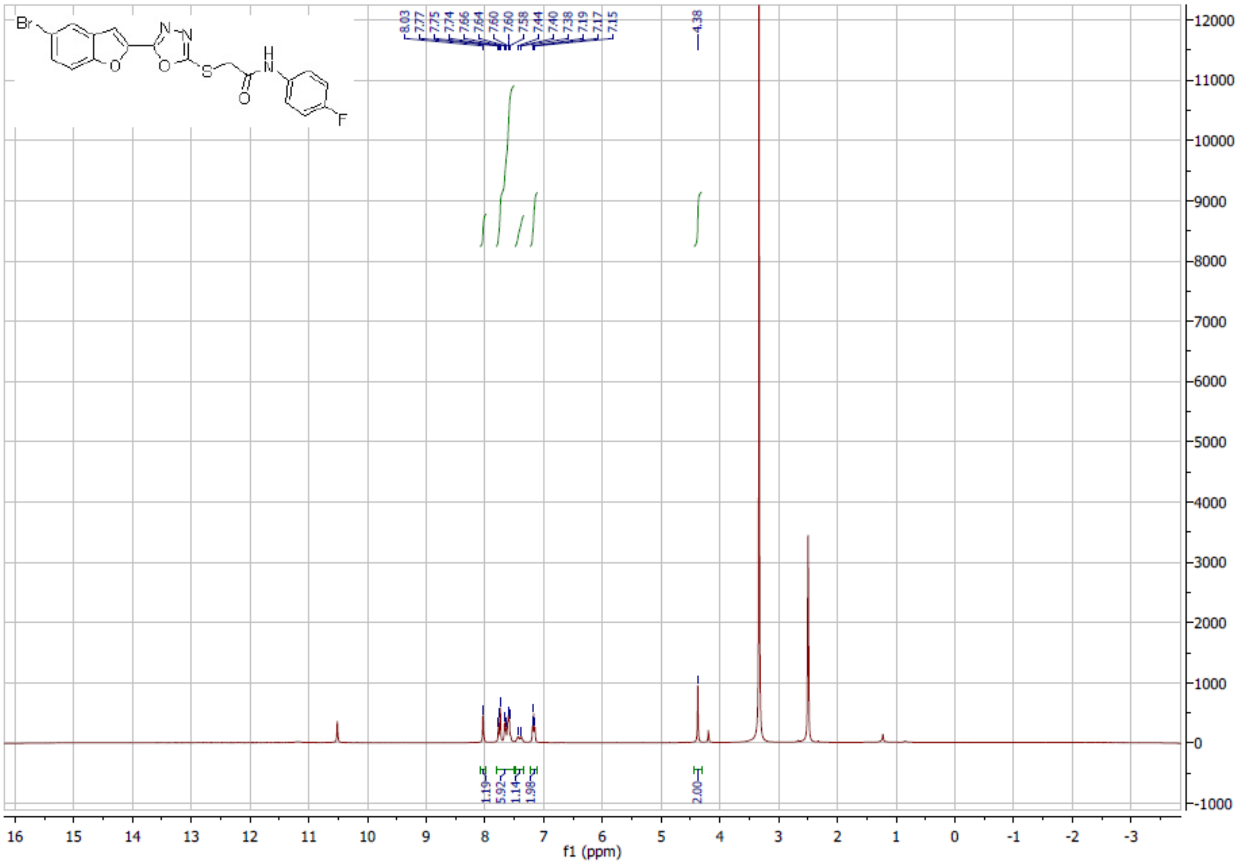

**Figure S7.**$^{1}$H NMR spectrum of compound **5d**

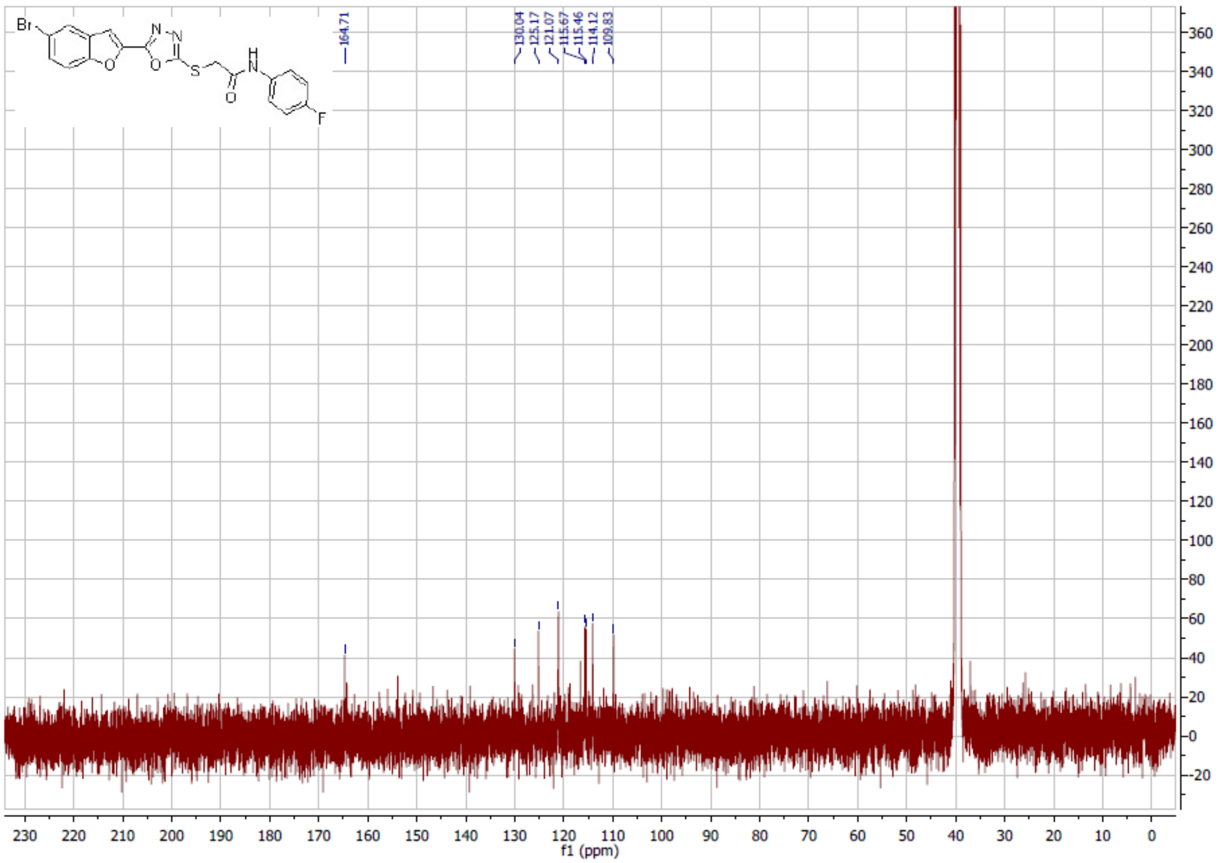


**Figure S8.** $^{13}$C NMR spectrum of compound **5d**

**2-((5-(5-bromobenzofuran-2-yl)-1,3,4-oxadiazol-2-yl)thio)-N-(3,4-dimethylphenyl)acetamide (5e)**

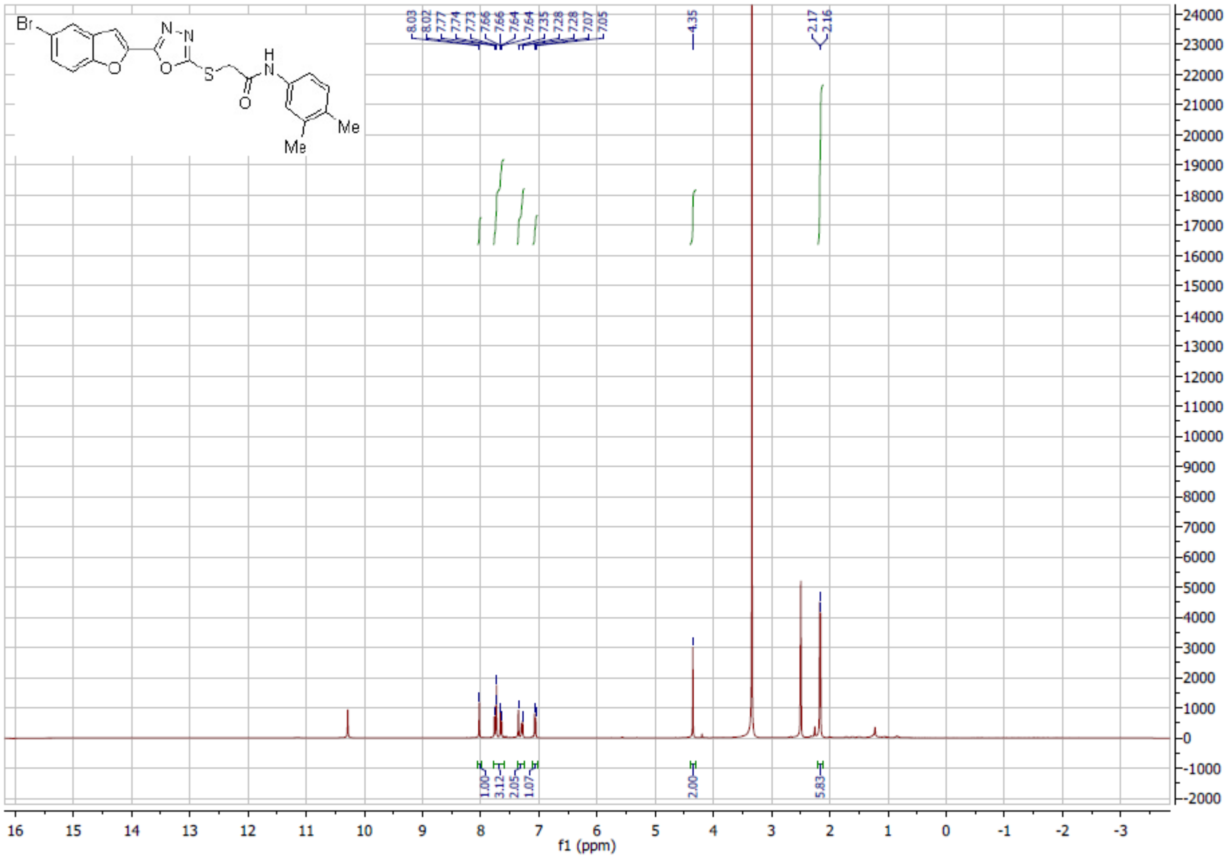


**Figure S9.**$^{1}$H NMR spectrum of compound **5e**

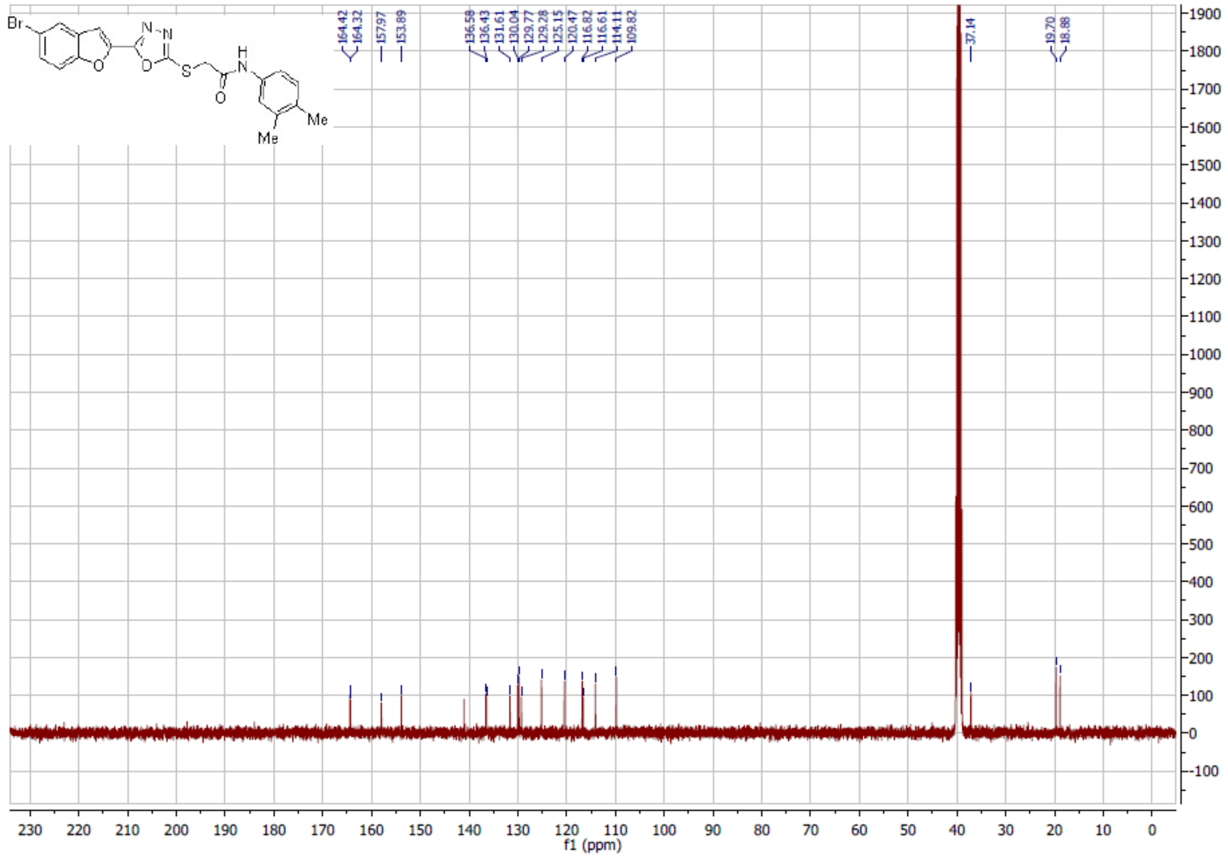

**Figure S10.**$^{13}$C NMR spectrum of compound **5e**

**2-((5-(5-Bromobenzofuran-2-yl)-1,3,4-oxadiazol-2-yl)thio)-N-(2-methoxyphenyl)acetamide (5f)**

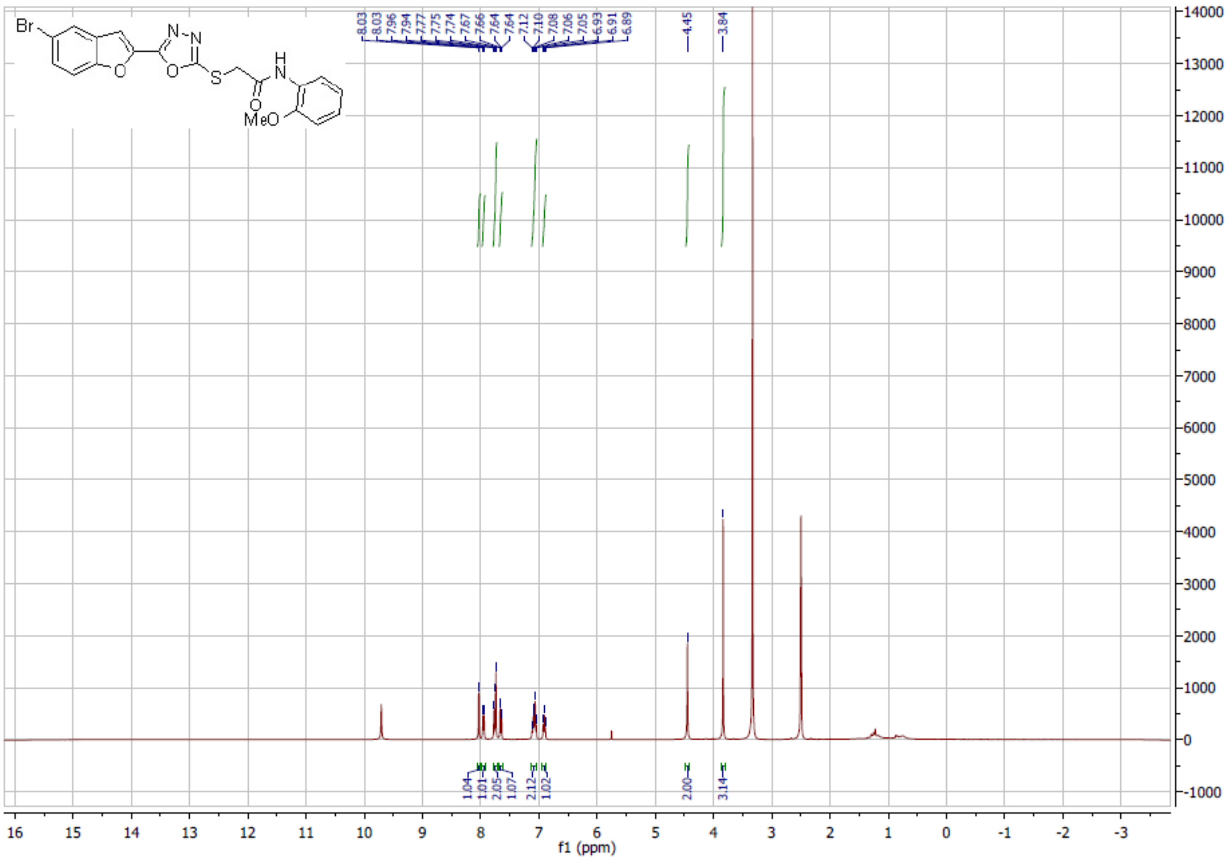

**Figure S11**$^{1}$H NMR spectrum of compound **5f**

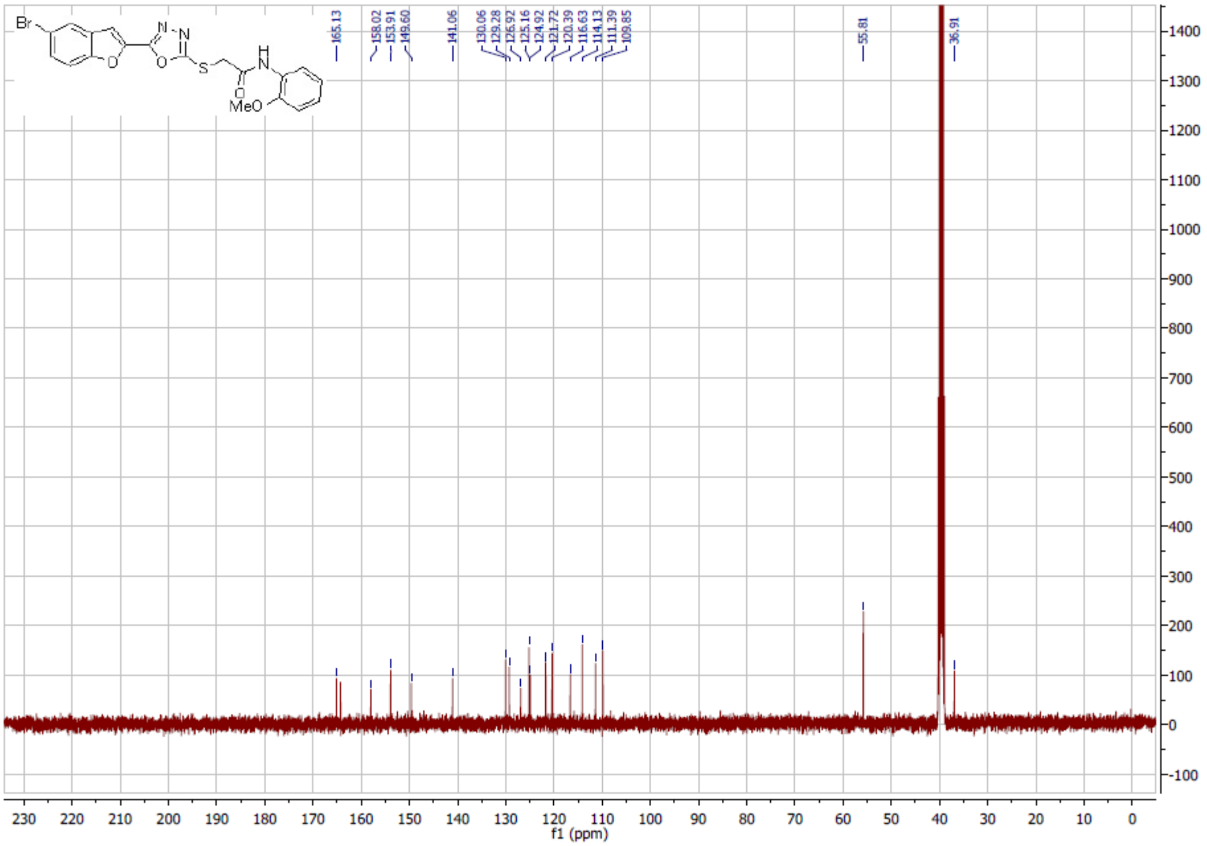


**Figure S12.**$^{13}$C NMR spectrum of compound **5f**

**2-((5-(5-Bromobenzofuran-2-yl)-1,3,4-oxadiazol-2-yl)thio)-*N*-phenylacetamide (5g)**

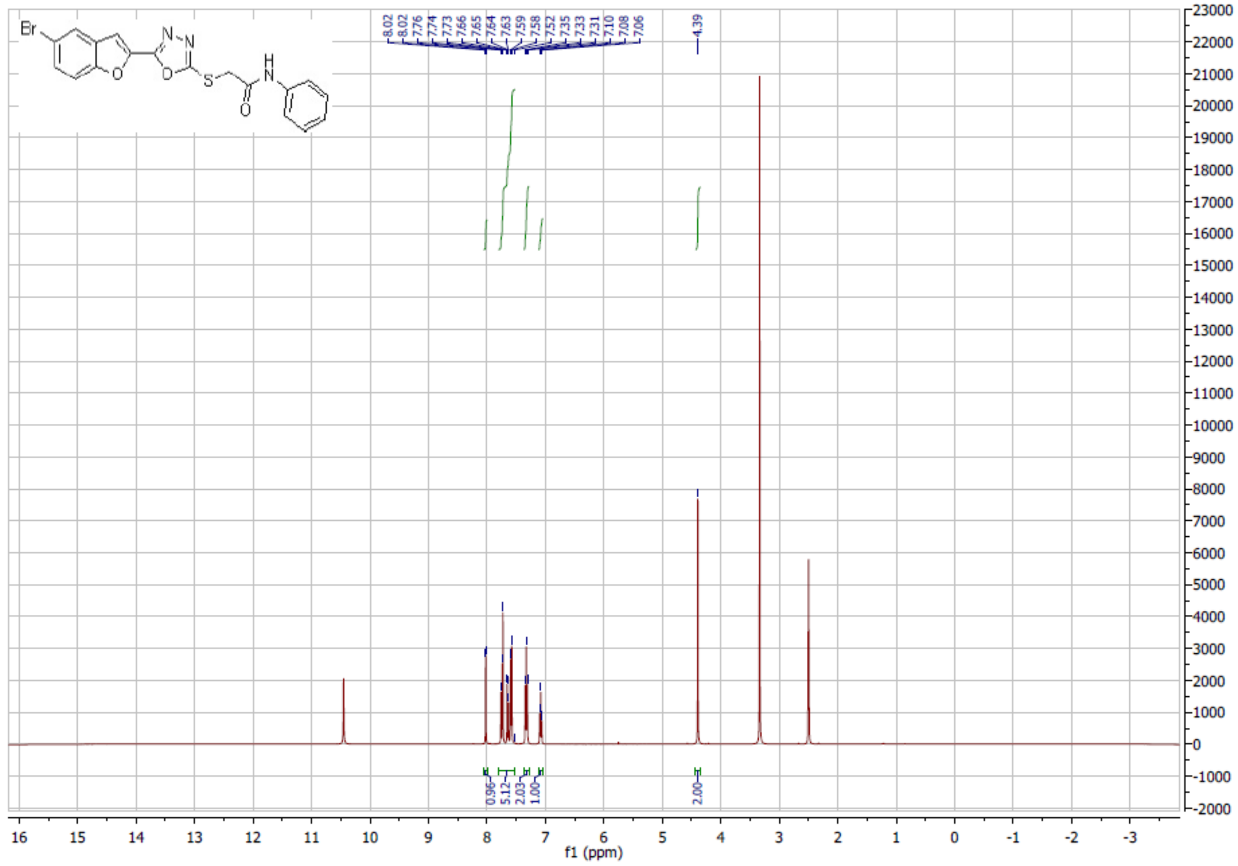

**Figure S13.** $^1$H NMR spectrum of compound **5g**

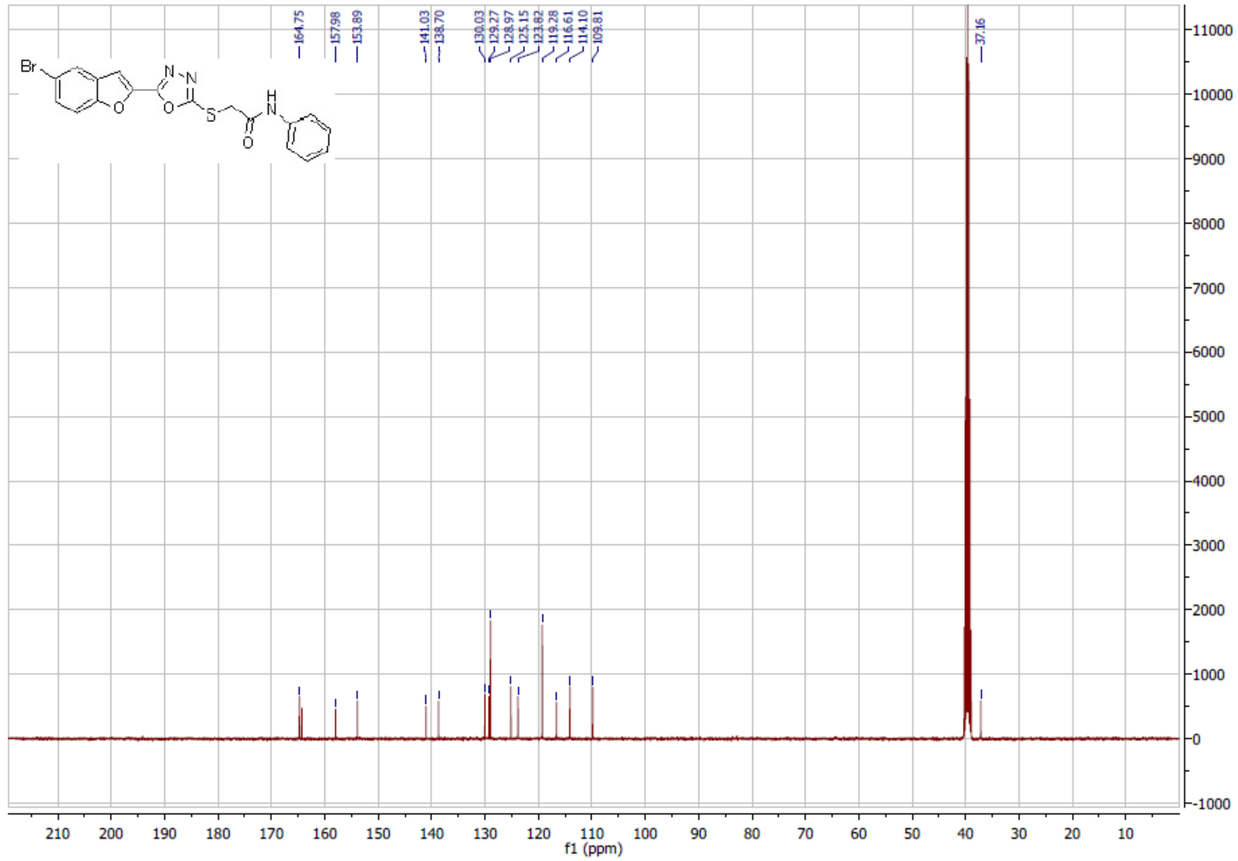


**Figure S14.**$^{13}$C NMR spectrum of compound **5g**

**2-((5-(5-Bromobenzofuran-2-yl)-1,3,4-oxadiazol-2-yl)thio)-*N*-(2,4-dimethylphenyl)acetamide (5h)**

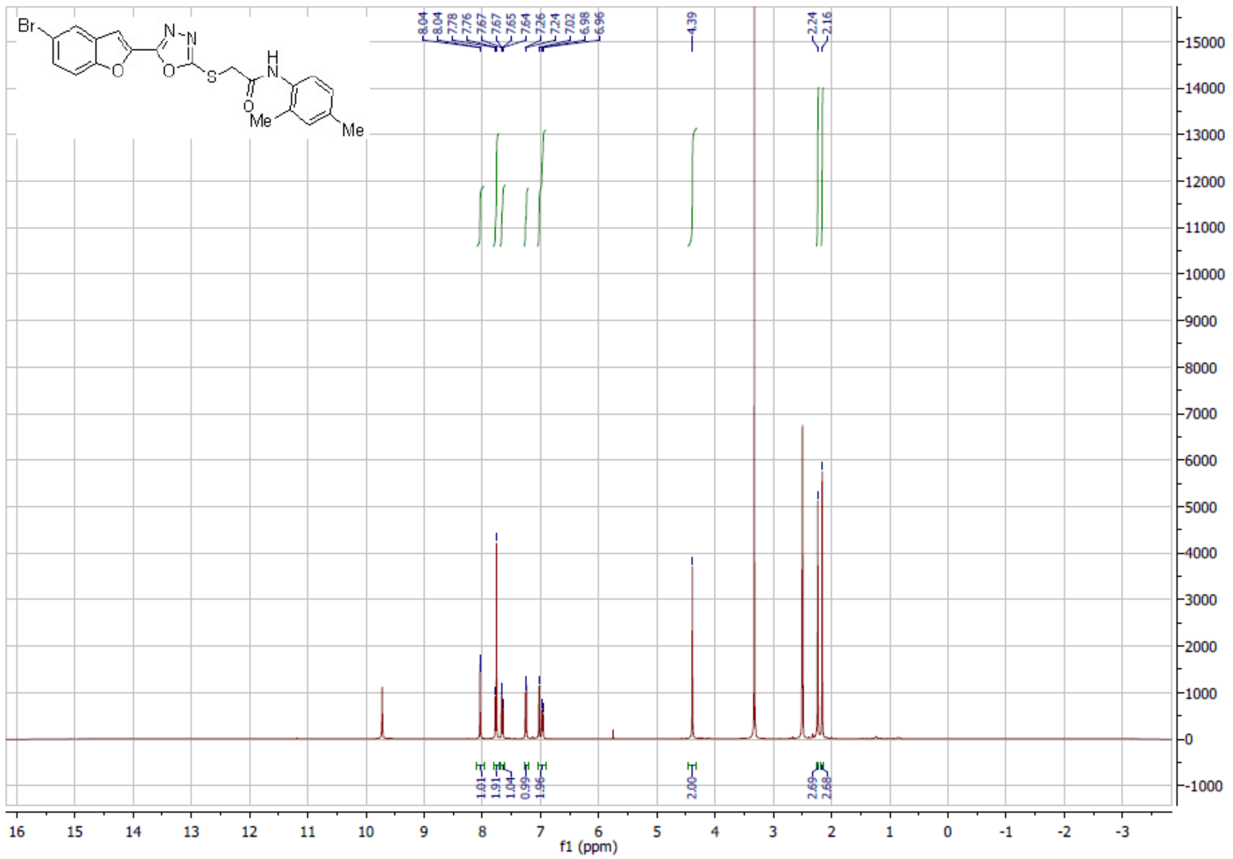

**Figure S15.**$^{1}$H NMR spectrum of compound **5h**

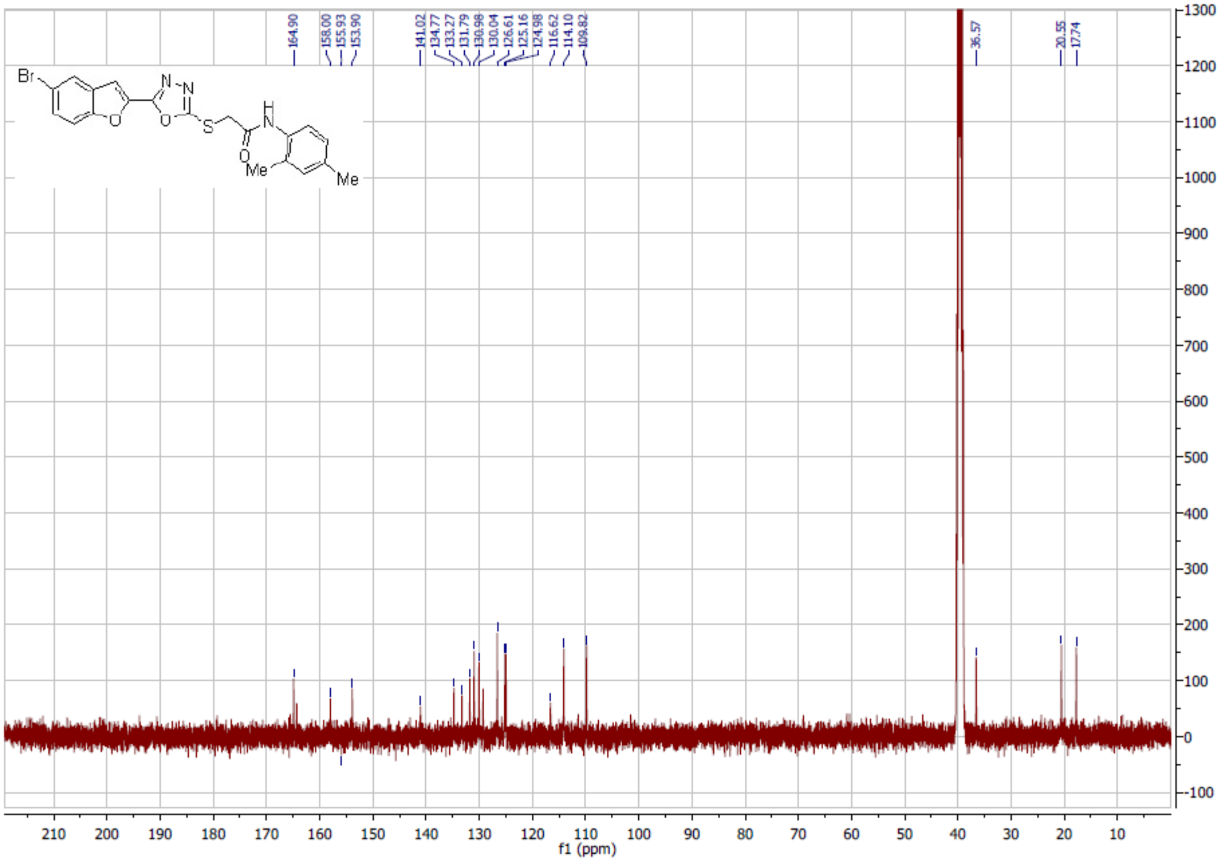


**Figure S16.**$^{13}$C NMR spectrum of compound **5h**

**2-((5-(5-Bromobenzofuran-2-yl)-1,3,4-oxadiazol-2-yl)thio)-N-(2,5-dimethoxyphenyl)acetamide (5i)**

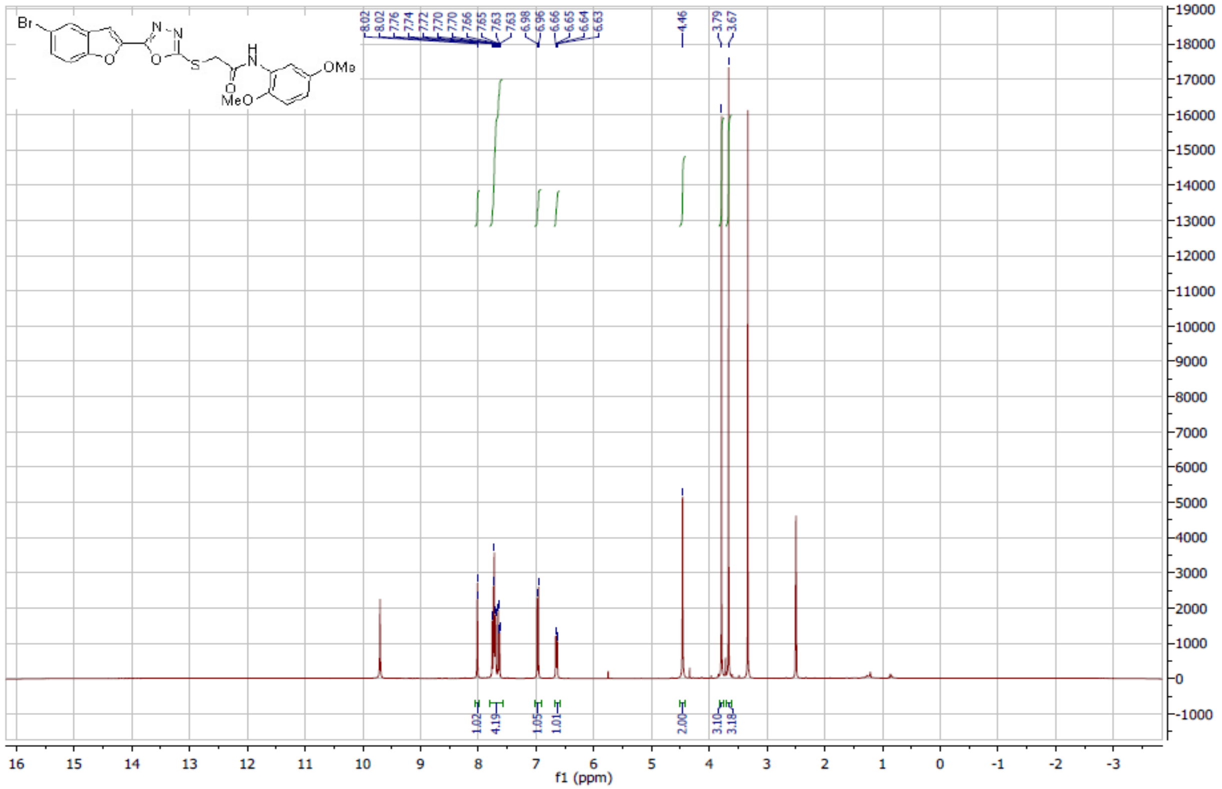

**Figure S17.**$^{1}$H NMR spectrum of compound **5i**

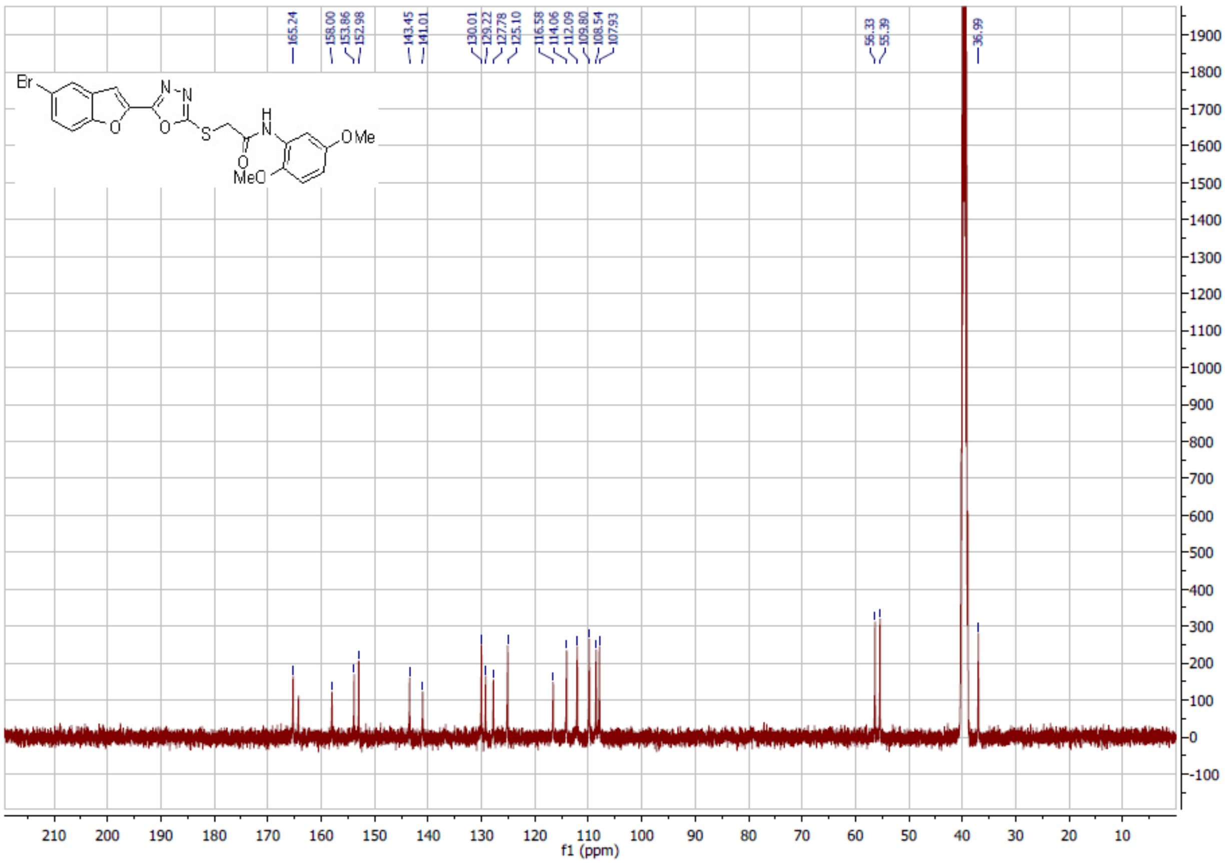


**Figure S18.** $^{13}$C NMR spectrum of compound **5i**

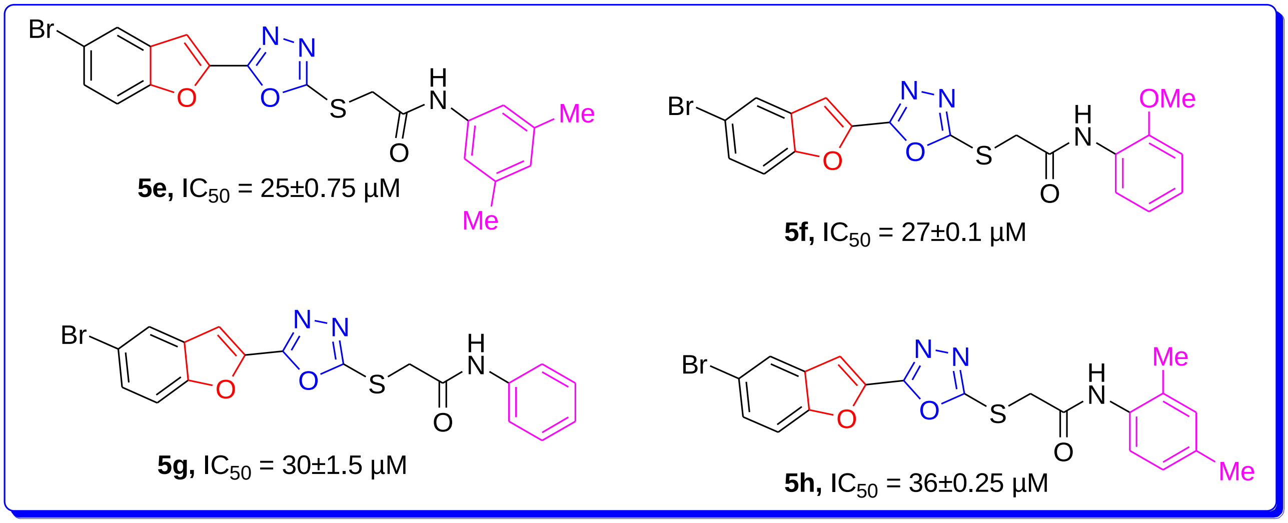


**Figure S19.** The moderate biologically active oxadiazole based furan derivatives **5e**- **5h** as tyrosinase inhibitors

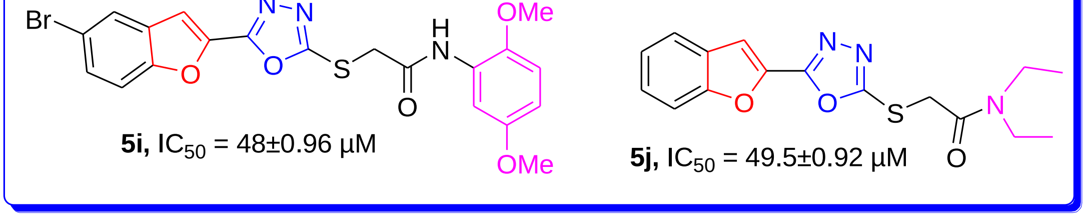


**Figure S20.** The least biologically active oxadiazole based furan derivatives **5i**- **5j** as tyrosinase inhibitors
